# Indigenous Knowledge and Quantitative Analysis of Medicinal Plants Used to Remedy Respiratory Tract Disorders in Mid-Western Tanzania

**DOI:** 10.1155/bmri/8534815

**Published:** 2024-11-28

**Authors:** David Sylvester Kacholi, Halima Mvungi Amir

**Affiliations:** Department of Biological Sciences, Dar es Salaam University College of Education, University of Dar es Salaam, PO Box 2329, Dar es Salaam, Tanzania

**Keywords:** ethnobotany, ethnopharmacology, fidelity level, medicinal plants, respiratory infections

## Abstract

This study is aimed at documenting the indigenous knowledge and quantitative analysis of medicinal plants (MPs) used by traditional health practitioners (THPs) of Urambo District in mid-western Tanzania to manage respiratory tract disorders (RTDs). The ethnomedicinal data were collected using semistructured interviews with 55 THPs using a snowballing technique in the district. The data were analysed for indigenous knowledge among gender, age groups, education status, and experience. Family importance value (FIV), use value (UV), relative frequency of citation (RFC), informant consensus factor (ICF), and Jaccard index (JI) were computed. A total of 42 MPs representing 28 families were recorded being used against RTDs in the district. Fabaceae was the dominant family in terms of species (16.7%) and FIV (84%). Decoction (51.0%) was the preferred technique for preparing remedies, while trees (61.9%) and leaves (38.1%) were the most utilised life form and plant parts, respectively. The RFC in the current study varied from 0.055 (*Musa paradisiaca* L.) to 0.655 (*Zingiber officinale* Roscoe) and 0.073 (*Dichrostachys cinerea* (L.) Wight & Arn.) to 0.673 (*Entada abyssinica* Steud. ex A.Rich.), respectively. The highest ICF was recorded for cough (0.922). The JI ranged from 2.7 to 7.9. Among the documented MPs, 55% had least concern, 2% were endangered, 7% had data deficiency conservation status, and 36% had no record in the IUCN Red List. The study revealed that the district's population depends on MPs for healthcare. Thus, conservation strategies are needed for the sustainable utilisation of the MPs. Importantly, the documented MPs hold immense potential in future pharmacological and phytochemical studies, offering hope for the development of new drugs for RTDs. Also, the study suggests the need for scientific validation of the MP's efficacy and safety.

## 1. Introduction

Since time immemorial, plants have been used to remedy various human and animal ailments, and they are considered vital in human healthcare [1]. Plants offer livelihood benefits, including timber, firewood, food, fodder, medicine, construction materials, and income [2, 3]. The medicinal plant (MP) knowledge has advanced across nations, cultures, and over time and space based on various medicinal systems. Moreover, each cultural society has a consociate and experience specific to its antique, cultural, and even spatial environment, representing an irreplaceable reservoir of skill and substantial potential for yet unexplored use of natural resources [4, 5].

Globally, respiratory tract disorders (RTDs) are a public health concern and account for over 10 million deaths. The most common RTDs include asthma, cough, flu, common cold, bronchitis, chronic obstructive pulmonary disease, pneumonia, tuberculosis, acute respiratory infections, and lung cancer [6, 7]. In Africa, particularly in Tanzania, cough, flu, colds, tuberculosis, pneumonia, and HIV-related RTDs are the most common respiratory conditions [8–10], whereby between 2006 and 2015, about 13% of all mortality in the country was due to RTDs [11]. Since 2020, the COVID-19 pandemic has triggered a heave of RTDs and become the foremost cause of morbidity and mortality [7, 12]. In rural areas like those of Urambo District, where modern health facilities are scarce, RTDs are widespread and put a burden on the locals' health and economy.

Nearly 80% of the human population in low- and middle-income countries still depend on MPs for their primary healthcare [13, 14]. Contemporary health facilities and associated services are habitually accessible only to a few people as the amenities are either too expensive or few health facilities are reachable for too many people [15]. Consequently, MPs remain the source of therapeutic care in most low- and middle-income countries [16]. In Tanzania, about 80% of the population lives in rural settings where modern health facilities are scarce; hence, they depend on MPs for their primary healthcare needs. The limited access to modern healthcare could result in suboptimal health outcomes for individuals in the study area. Moreover, the rampant usage of MPs by rural inhabitants could also be associated with the accessibility, efficacy, cultural acceptability, and affordability of the medication [17, 18]. Due to socioeconomic realities and the absence of enough contemporary health facilities, the locals in the rural areas of Urambo District bank to MPs for their primary healthcare needs, including management of RTDs.

Although previous studies have explored the MPs in Urambo District over the last two decades [17, 19–21], little is known about MPs used to remedy RTDs within the district. Therefore, this study is aimed at documenting indigenous knowledge and quantitatively analyzing MPs used by the local traditional health practitioners (THPs) in Urambo District to treat RTDs. Moreover, the study checked the conservation status (CS) of the recorded MPs in the International Union for Conservation of Nature (IUCN) Red List of Threatened Species [22].

## 2. Materials and Methods

### 2.1. Study Area

**Figure 1:**
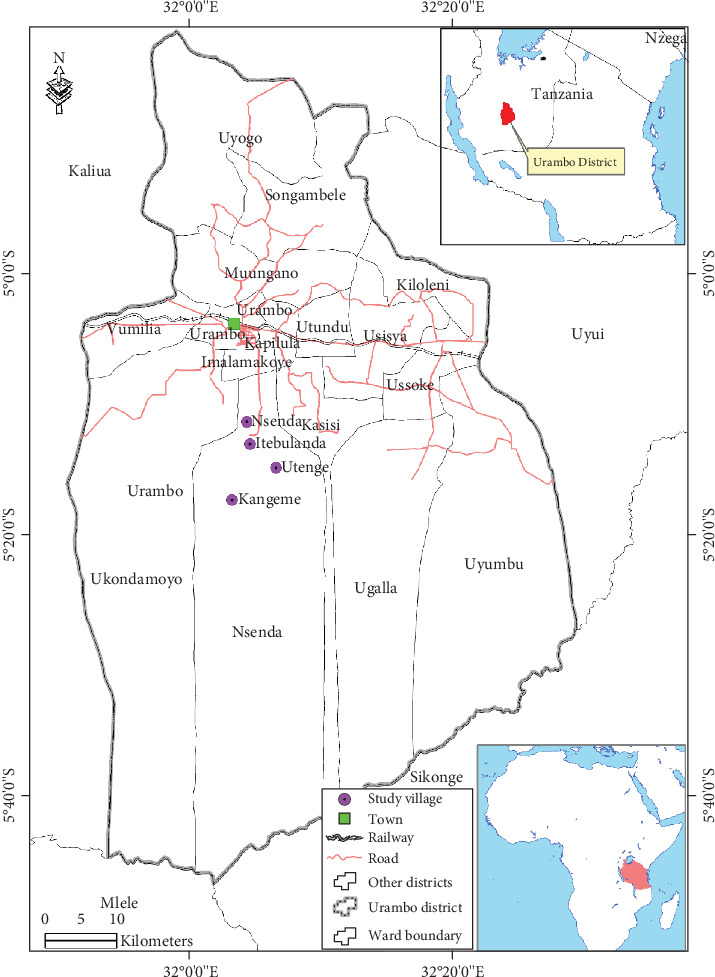
Map showing the study areas and the setting of the district in Tanzania.

This study was conducted in Urambo District, lying at latitude 04°41⁣′ to 05°44⁣′ S and longitude 31°51⁣′ to 32°26⁣′ E in the Tabora region, mid-western Tanzania. The district is one of the seven districts in the Tabora Region. It is bordered by the Uyui District in the east, Kaliua District in the north, Mlele District in the southwest, and Sikonge District in the southeast (Figure 1). Urambo District lies 1000 and 1800 m above the mean sea level, covering an area of 5416 km^2^. It has a population of 192,781 and an average household size of 5.9. The annual mean population growth rate of the district is 2.9%. The area's climate is bimodal; the dry season lasts from May to October, and the rainy season lasts from November to April. The mean annual rainfall of the study area is 1370 mm [21]. Local dwellers in the district, who are principally the Nyamwezi tribe, rely solely on agriculture and livestock keeping for their livelihood. The primary food and cash crops cultivated in the district are rice and tobacco. The Nyamwezi are well-known in the country for their traditional healing practices [17, 21]; hence, the district's choice was due to their excellent conventional knowledge of MPs.

### 2.2. Ethnobotanical Data Collection

**Figure 2:**
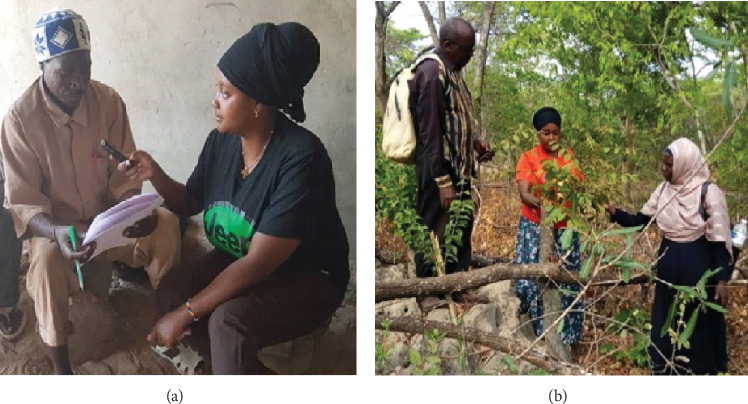
(a) Interview with traditional health practitioners and (b) conducting a field walk.

This ethnomedicinal study of MPs for respiratory *disorders* was conducted from August 2020 to March 2021 in Urambo District. A total of 55 THPs were involved in this study. A snowballing sampling method was used to get the THPs in the communities, where 17 THPs were from Utenge ward, 13 were from Itebulanda, 13 were from Kangeme, and 12 were from Nsenda ward. The aim of the study was communicated to all THPs, and verbal informed consent to participate in the study was requested before the interview. The THPs who agreed to participate were interviewed using a semistructured interview and field walk (Figure 2). The dialogues with THPs were done in Swahili. During the survey, demographic profiles of THPs (gender, age, education level, experience, and residence) and MPs (plant name, treated ailment, plant parts used, mode of preparation, and route of administration of remedies) were gathered (Table 1). The identification of MPs was done straight away in the area by a skilled and qualified botanist, Mr. Shaaban Makaka. The MPs that could not be identified in the field were collected and pressed for further identification in the College Herbarium (DUCE Herbarium). The collected MPs were identified, and their scientific names were verified, following the Flora of East Africa [23] and Plants of the World Online database (https://powo.science.kew.org/).

### 2.3. Research Clearance and Ethical Approval

The office of the Vice Chancellor of the University of Dar es Salaam provided research clearance and ethical approval to undertake this study.

### 2.4. Quantitative Data Analysis

#### 2.4.1. Demographic Profile and Indigenous Medicinal Knowledge

The sociodemographic data were descriptively analysed for significant differences within variables using one-way ANOVA and independent *t*-test. All analyses were carried out using Microsoft Excel 2010 and QED statistics software.

#### 2.4.2. Relative Frequency of Citation (RFC)

The RFC index demonstrates the traditional importance of each MP in the study area [24]. RFC value ranges from 0 (where no one cites MP as useful) to 1 (when all informants cite an MP as useful). The index does not consider the reports and was computed as per the equation shown below.

(1) $$\begin{array}{c} \text{RFC}=\frac{\text{FC}}{N}\left(0<\text{RFC}<1\right) \end{array}$$

where FC is the frequency of citation (the number of informants citing the use of a particular MP) and *N* is the total number of informants involved in the study.

#### 2.4.3. Family Importance Value (FIV)

The FIV is a quantitative measure that analyses the importance of the botanical family as per the informant. It was calculated using the following formula [25].

(2) $$\begin{array}{c} \text{FIV}=\frac{\text{fc}}{N}\times 100 \end{array}$$

where fc is the number of informants reporting the family and *N* is the total number of informants involved in the study.

#### 2.4.4. Use Value (UV)

The UV is an index that shows the relative significance of the MPs used by the locals in the study area. It is calculated using the formula shown below [26].

(3) $$\begin{array}{c} \text{UV}=\frac{\sum \text{U}}{N} \end{array}$$

where *U*  is the number of use reports cited by each informant for a given MP and *N* is the total number of informants.

#### 2.4.5. Plant Part Value (PPV)

The PPV index is used to identify the most utilised plant part in a medicinal formulation. The plant part with the highest PPV is the most exploited by the locals for remedy formulation compared to those with low values. The index was calculated using the formula below [27].

(4) $$\begin{array}{c} \text{PPV}=\frac{\sum {\text{RU}}_{\left(\text{plant part}\right)}}{\sum \text{R}\text{U}}\times 100 \end{array}$$

where RU_(plant part)_ is the sum of uses cited per plant part and RU is the number of use reports for all plants.

#### 2.4.6. Informant Consensus Factor (ICF) Ratio

The ICF is the most preferred quantitative method for identifying the potentially effective MP in a specific ailment category. Before computing the ICF, ailment categories were formed. Usually, the ICF values range from 0.00 to 1.00. The high ICF values imply that MPs are more pharmacologically active than those with low ICF values [28]. The index was calculated using the following formula:

(5) $$\begin{array}{c} \text{ICF}=\frac{N_{\text{ur}}-N_{t}}{N_{\text{ur}}-1} \end{array}$$

where *N*_ur_ is the number of use reports for a particular ailment category and *N*_*t*_ is the total number of MPs cited by informants in the ailment category.

#### 2.4.7. Jaccard Index (JI)

The JI was computed to compare the similarity of MPs between data from the present study and ethnomedicinal studies on RTDs already published in other African countries. This index is based on the presence or absence of species on each list and was calculated as follows [29]:

(6) $$\begin{array}{c} \text{JI}=\left(\frac{c}{a+b+c}\right)\times 100 \end{array}$$

where *a* is the total number of MPs of the present study, *b* is the total number of MPs of another ethnomedicinal study, and *c* is the number of MPs common to both studies.

#### 2.4.8. CS

The CS of the reported MPs growing in the wild was assessed. The data was documented for different conservation attributes following the IUCN. The recorded MPs were categorised based on the IUCN Red List categories and Criteria 2022 [22].

## 3. Results and Discussion

### 3.1. Demographic Profile and Indigenous Knowledge

Among 55 interviewed THPs, 81.8% were males, and 18.2% were females (Table 2). Statistical analysis revealed no difference between genders regarding MPs knowledge (*t* = 1.761, *p* = 0.084). The present finding indicated that the belief that females know more MPs because they are homemakers and are responsible for the family's health or men know more due to time spent dealing with natural resources cannot permanently be generalised as both have the same rich ethnomedicinal knowledge and no gender-based pattern of expertise. A similar observation was also reported in Brazil [30].

In terms of age, most THPs were of the age group of 21–40 years (47.3%), followed by the age group 41–60 years (34.5%), while the remaining two age groups (< 20 years and > 60 years) constituted 18.2% (Table 2). The findings showed that older generations had significantly higher ethnomedicinal knowledge than the younger generations (*p* ≤ 0.001). The older generation has accrued more ethnomedicinal knowledge through serving in the field and interacting with Mother Nature longer than the younger informants. Similar results were reported in other ethnomedicinal studies conducted within the country [31] and from other countries [32]. The lower ethnomedicinal knowledge exhibited by younger generations may be attributed to urbanization, modernization, and a shift towards Western medicine, highlighting a potential loss of cultural heritage [33, 34]. Similarly, the decline of ethnomedicinal knowledge among younger generations has been reported in other countries like Mozambique [35] and Indonesia [36], where the young are more interested in contemporary medicine than traditional remedies. The finding communicates that conventional medicinal knowledge is in peril, as oral transmission from the older to the more youthful generation is no longer reliable. Thus, recording and archiving MPs of significant use for treating ailments is paramount.

Regarding education level, 80% of the THPs had primary education, followed by illiteracy (12.7%), secondary education (5.5%), and tertiary education (1.8%) (Table 2). The statistical difference between education level and ethnomedicinal knowledge was significant (*p* ≤ 0.001), indicating that MPs' knowledge and use decline with increasing education level. The current findings agree with a similar study conducted in Morocco, Rif [27], and Nigeria [32].

In terms of healing experience, most THPs had 5–10 years (40%) in the sector, and the THPs with greater than 15 years revealed to have significantly higher knowledge of MPs, followed by those with 11–15 years, 5–10 years, and lastly THPs with less than 5 years (Table 2). The findings suggest that the THPs who have worked for a long time in the sector have accumulated more experience and knowledge in utilising the MPs. Furthermore, the acquisition of MPs knowledge was primarily acquired from close family members (67.3%), herbalists (23.6%), and divine and supernatural powers (9.1%), similar to an ethnomedicinal study from Kenya [37].

### 3.2. MP Diversity

A total of 42 MPs belonging to 40 genera of 28 botanical families were documented with traditional medicinal uses against RTDs (Table 3). The most encountered botanical families were Fabaceae (7 species, 16.7%); Myrtaceae (3 species, 7.1%); and Apocinaceae, Euphorbiaceae, Loganiaceae, Phyllanthaceae, Rubiaceae, and Rutaceae (with 2 species, 4.8% each), while the remaining 20 families were each represented by one species. The findings concerning the prevalence of the utilisation of family Fabaceae in the district agreed with other ethnobotanical studies conducted in the district [21, 23] and other parts of the country [38]. The dominance of the Fabaceae in ethnomedicinal use against respiratory ailments was also reported in Ethiopia [39], Kenya [40], Zimbabwe [41], and South Africa [42]. The use of MPs to manage disorders that belong to registered families could be influenced by the culture and availability of MPs in a geographical setting [43]. Moreover, it could probably be due to an extensive range of distribution, as well as the production of potential secondary metabolites with effective antimicrobial activities against RTDs.

### 3.3. Therapeutic Significance

**Figure 3:**
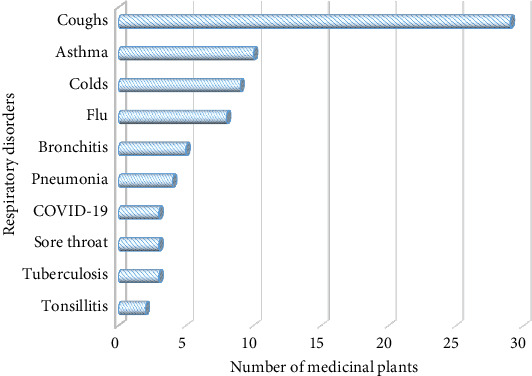
Number of medicinal plants for each respiratory tract disorder.

According to the current survey, 10 RTDs were reported by the THPs being commonly treated by MPs (Figure 3). The most reported RTDs regarding MPs were cough (29 species, 69.0%), followed by asthma (10 species, 23.8%), colds (9 species, 21.4%), and flu (8 species, 19.0%). The remaining RTDs were treated with less than six MPs. Similar studies in Pakistan [25], Palestine [44], Ethiopia [39], and Kenya [40] reported cough, asthma, and colds being the common RTDs treated by many MPs, showing their prevalence as RTDs across many realms. The wealth of traditional remedies could also be associated with the prevalence of the disorders.

### 3.4. Origin and Life Forms

**Figure 4:**
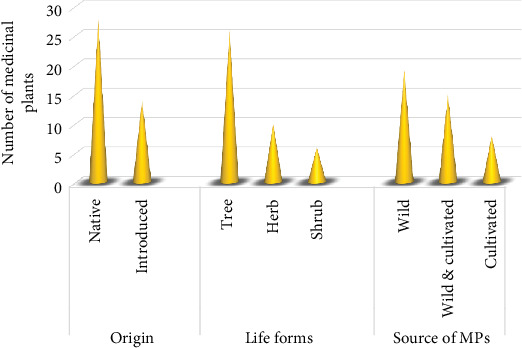
Origin, life form, and sources of medicinal plants.

In the present survey, 66.7% of the MPs were native to Tanzania, and 33.3% were introduced from elsewhere. Among the reported MPs, tree (61.9%) was the most used life form, followed by herbs (23.8%) and shrubs (14.3%) (Figure 4). The frequent use of trees for medicinal purposes was also reported in other ethnobotanical studies within the country [17, 45]. Still, the current finding differs from other studies in Pakistan [25] and Ethiopia [39], which reported herbs to be dominant in treating respiratory disorders. The wide use of trees by the THPs in the district is due to their dominance and availability throughout the year compared to other life forms, which are affected by seasonality [46]. Moreover, the frequency of tree use indicates that the local THPs have rich indigenous knowledge of using the life form for respiratory disorders.

### 3.5. Sources of MPs

About 45.2% of the MPs were exclusively collected from the wild, 35.7% were from both wild and cultivation, and 19.1% were solely gathered from cultivation (Figure 4). Similarly, some ethnomedicinal studies in Tanzania [17, 38, 45] and other African countries [39, 40, 47] showed that most MPs are soured from wild environments. The high utilisation of MPs in wild environments is described by the fact that MPs that grow in the wild are believed to be rich in bioactive compounds [48]. Overharvesting of wild MPs for medicinal purposes can lead to depletion of natural resources and threaten biodiversity, especially, if proper conservation measures are not implemented.

### 3.6. Plant Parts Used for Remedy Formulation

**Figure 5:**
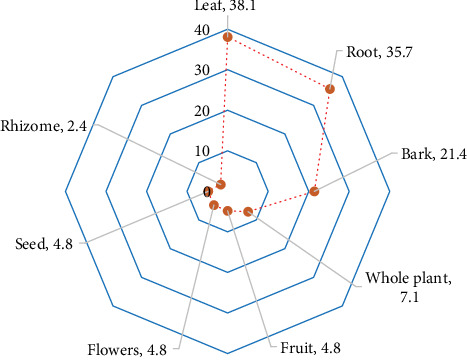
Radar diagram showing plant part values (PPVs) for each medicinal plant part.

The PPV index helps understand the principal MP part used commonly for medicinal formulations in a local area. In this study, leaves were the most preferred plant part with a PPV of 38.1%, followed by roots (35.7%); bark (21.4%); whole plant (7.1%); fruits, flowers, and seeds (each with 4.8%); and rhizome (2.4%) (Figure 5). Likewise, studies conducted in different parts of the world [39, 42] showed leaves to be the commonly preferred MP part for managing RTDs. The preference for leaves could be related to their availability and ease of harvest compared to other plant parts and the preparation and synthesis of abundant bioactive ingredients, which are pharmacologically active against various disorders [49, 50]. Moreover, it could be attributed to their renewal potential and collection that does not jeopardise the existence of parent MPs over a period.

### 3.7. Modes of Preparation and Routes of Administration of Remedies

**Figure 6:**
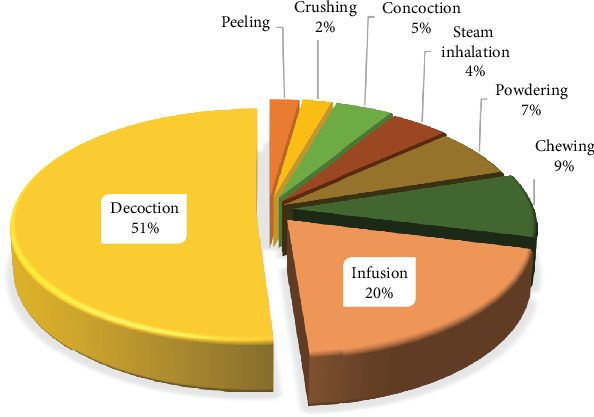
Modes of preparation used in the formulation of remedies.

The RTD remedies are prepared depending on the MP life form and the part used. Most traditional recipes are formulated with or without a subsidiary substance. In the present study, decoction (51.0%) and infusion (20.0%) were the commonly preferred methods for preparing remedies against RTDs. Other forms accounted for 29.0% (Figure 6). The decoction involves boiling MP material in water to speed up the extraction of the required bioactive constituents [51], but it also detoxifies and sterilises the used plant materials. Water is the commonly used solvent because it is affordable and more efficient for extracting soluble metabolites than other solvents [23]. Moreover, additives like sugar, honey, milk, and butter are used to intensify the efficacy and potency of the medicines, making the medication tasty and evading any intestinal disquiet [52]. Most RTDs (95.2%) were administered orally, while 4.0% were through steam inhalation. The mode of administration depends on the physical condition of the patient and the stage of the ailment. Comparable findings have also been reported in similar ethnomedicinal studies on RTDs conducted elsewhere [25, 44].

### 3.8. Quantitative Analysis of Ethnorespiratory Information

#### 3.8.1. FIV

To evaluate the most significant plant families in the study area, the FIV was computed as per Equation (2), and each family's values are presented in Table 3. The FIV ranged from 5% to 84%. Fabaceae displayed the supreme FIV (84%), followed by Zingiberaceae (66%) and Myrtaceae (62%). The dominance of Fabaceae could be attributed to its richness in terms of MPs in the area, as it is foremost in most lowland areas in the country [53], and familiarity of the family in ethnomedicine. Moreover, the finding shows the importance of individual MPs in the area and the popularity of a plant family, which could be associated with MPs' availability and their connection with traditional remedies [54]. Additionally, the low FIVs exhibited by some families, such as Musaceae (5%) and Solanaceae (7%), could signify less availability of the MP species in the area and less familiarity among locals about managing RTDs [55].

#### 3.8.2. RFC

The RFC is used to assess frequently utilised MPs for managing different ailments by the locals in an area. The RFC in the current study varied from 0.055 to 0.655. The MPs with the highest RFC were *Z. officinale* (0.655), followed by *E. abyssinica* and *Cassia abbreviata* Oliv. (with 0.527 each), and *Citrus limon* (L.) Burm.f (0.473). The MPs with higher RFC values indicate that they are very familiar to the majority of THPs in treating RTDs [56]. Hence, they must be further pharmacologically and phytochemically investigated to recognise their bioactive ingredients for treating RTDs.

#### 3.8.3. UV

The UV index demonstrates the comparative importance of useful MPs. The information offered by the UV index is essential for ethnomedicinal research as it shows the utmost harvesting pressure and provides a new avenue for drug discovery. The UV could efficiently assess the most useful MPs or group of MPs to a specific society group and consider potential uses within the group [57]. The present study revealed that UV varied to overcome coughs and colds. *E. abyssinica* had a higher UV (0.673), followed by *Syzygium aromaticum* (L.) Merr. & L.M.Perry (0.655) and *Z. officinale* (0.618). The findings show that the THPs have good indigenous knowledge of the ethno MPs and practices. The MPs with high UV must be further examined for phytochemical and pharmacological to diagnose their bioactive elements that can be used to develop modern drugs [58]. Likewise, these MPs should be prioritised for conservation as their preferential consumption may compromise their survival due to overharvesting.

**Figure 7:**
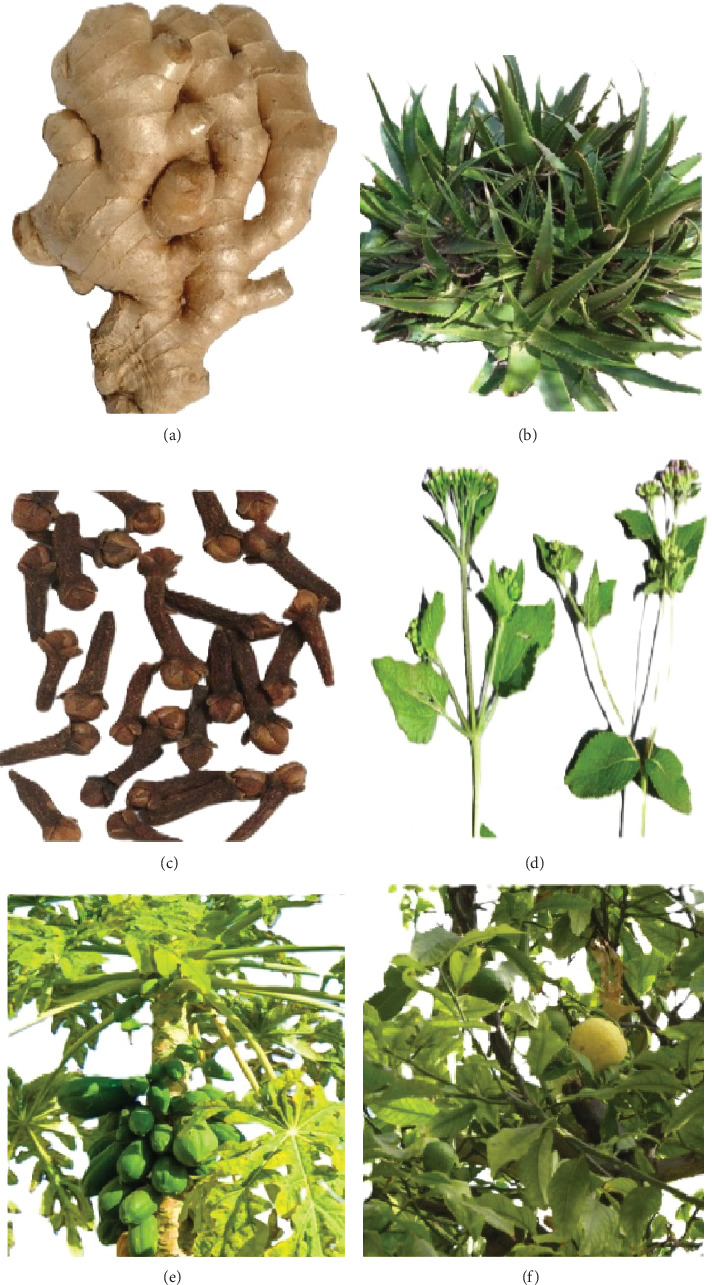
Some medicinal plants used by THPs in treating respiratory transmitted disorders in Urambo District: (a) *Zingiber officinale*, (b) *Aloe vera*, (c) *Syzygium aromaticum*, (d) *Ageratum conyzoides*, (e) *Carica papaya*, and (f) *Citrus limon*.

Extracts of stem bark and roots of *E. abyssinica* are reported to treat respiratory bacterial infections due to the passion of quercitrin, a glycoside moulded from the flavonoid quercetin and rhamnose, with an MIC of 3.12 *μ*g/mL against *Salmonella typhimurium* [59]. *Z. officinale* is a popular MP used against RTDs in different parts of the globe, such as in Indonesia [57], Ethiopia [60], and Nigeria [61]. The MP is well known for the possession of abundant active constituents, such as phenolic compounds (gingerols, shogaols, and paradols) and terpene compounds (*β*-bisabolene, *α*-farnesene, *α*-curcumene, zingiberene, and *β*-sesquiphellandrene) [62]. Other MPs reported in this study but reported elsewhere for RTDs include *C. abbreviata* in Tanzania [59]; *Ageratum conyzoides* L. in Indonesia [57]; *Mangifera indica* L., *Citrus aurantifolia* (Christm.) Swingle in Pakistan [25]; and *Clerodendrum myricoides* (Hochst.) R.Br. ex Vatke., *Psidium guajava* L., *Eucalyptus globulus* Labill., *Solanum incanum* L., *C. limon*, and *C. aurantifolia* in Ethiopia [60]. Some MPs used for treating RTDs are shown in Figure 7.

#### 3.8.4. ICF

All MPs used for managing RTDs were grouped into 10 ailment categories (Table 4). The ICF values ranged from 0.714 to 0.992. The high pervasiveness of the ailments above may echo the poor socioeconomic and hygienic conditions of the deprived people. The findings agree with other ethnomedicinal studies conducted in Ethiopia [60]. The highest ICF was recorded for cough (0.922), followed by flu (0.919) and asthma (0.910). The lowest ICF was recorded for tuberculosis (0.714). The higher ICF value suggests a high degree of agreement among THPs and the usage of diverse MPs for the management of a particular ailment, while the low ICF value is vice versa. Furthermore, low ICF may signal the presence of fewer incidences of a specific category of illness in the study area or designate less reliability of the informer's knowledge [49]. High ICF values could also be related to the high UV of MPs for a particular ailment category [63]. Thus, MPs with multiple uses in managing various ailments are believed to be effective medicine.

#### 3.8.5. Comparative Study of MPs Used for RTDs

A comparative analysis of MPs used for managing RTDs in Urambo District and seven African countries was conducted. The JI index ranged from 2.7 to 7.9 (Table 5). The high degree of similarity (JI = 7.9) was with the studies from the Maritime region in Togo [64], followed by Osun State in Nigeria [65]; Maputaland in South Africa [66]; and Kamuli, Kisoro, and Nakapiripirit districts in Uganda [16] with JI values of 7.9, 5.8, 5.3, and 5.1, respectively. The lowest similarity index was with Oum Rbai in Morocco, with a JI value of 2.7 (Table 5). Among the common MPs, six, namely, *Carica papaya* L., *C. aurantifolia*, *C. limon*, *Z. officinale*, *E. globulus*, *and A. conyzoides,* had a higher citation frequency among the compared studies.

#### 3.8.6. CS

**Figure 8:**
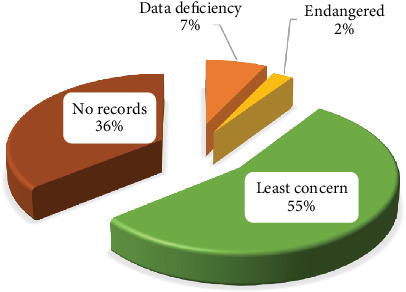
Conservation status of the recorded medicinal plants in Urambo District.

The unwarranted collection of MPs for timber, fodder, fuelwood, food, and other commercial manipulations has provided them with countless amounts of vulnerability [29]. The CS of all documented MPs was verified using the IUCN Red List of Threatened Species (IUCN 2022). In total, 36% (15 MPs) of the documented MPs had no record in the IUCN list. In comparison, 55% (23 MPs) were found to have the least concern (LC) CS (Table 3), which endorses an adequately stable population for the MPs at the global level. Additionally, one species (2%), namely, *C. myricoides*, was found to have an Endangered status, and three MPs (7%), namely, *Z. officinale*, *M. indica*, and *C. papaya* were found to have data deficiency status (Figure 8). From the above observations, this study believes that the conservation of the MPs should be fortified, and the CS of the MPs that have no records should be assessed.

## 4. Conclusion

This study revealed that the THPs in Urambo District are rich in indigenous knowledge and that a diversity of MPs is used to manage RTDs. The frequently treated RTDs were cough, asthma, colds, and flu. The older generation is revealed to possess an immense traditional knowledge of MPs compared to the younger generation. Thus, the traditional healing practice and its knowledge transfer in the district are at risk and require immediate conservancy before its extinction. This study offers baseline information to pharmacologists and phytochemists for the development of new drugs. This work can indeed be used to promote cultural conservation, MP conservation, and sustainable practices in the district, country, and globally. Moreover, this study suggests the need for scientific validation of MP efficacy and safety because relying solely on anecdotal evidence without rigorous scientific studies can lead to ineffective treatments or even harm to patients.

## Figures and Tables

**Table 1:** A questionnaire used during ethnobotanical data collection in Urambo District.

**Parameter**	**Information**
Informants	ID

Particulars	Gender (male/female)
	Age (years)
	Education level
	Experience (years)
	Residence/location
	Source of traditional healing practice

Questions	Which plants have you used for respiratory disorders? (Local name)
	Which ailment does each plant treat?
	Which plant part do you use for remedy formulation? (Root, leaf, bark, etc.)
	How is it used? (Fresh or dried)
	How do you prepare the remedies? (Infusion, topical, decoction, tea, etc.)
	How is each remedy administered? (Oral, topical, etc.)
	Where do you collect medicinal plant resources? (Wild or cultivated areas)

**Table 2:** Demographic profile of THPs and their indigenous knowledge.

**Parameter**	**Category**	**THPs**	**Percentage (%)**	**Mean ** **M** **P** **s** ± **SD**	**p** ** value**
Gender	Male	45	81.8	7.3 ± 0.6	0.084
	Female	10	18.2	6.9 ± 0.4	

Age groups (years)	< 20	3	5.5	5.9 ± 0.8	0.001
	21–40	26	47.3	6.1 ± 0.5	
	41–60	19	34.5	8.8 ± 0.7	
	> 60	7	12.7	9.3 ± 0.3	

Educational level	Illiterate	7	12.7	8.9 ± 0.7	0.001
	Primary	44	80.0	7.7 ± 0.4	
	Secondary	3	5.5	5.9 ± 0.3	
	Tertiary	1	1.8	3.1 ± 0.6	

Experience (years)	< 5	10	18.2	3.7 ± 0.9	0.001
	5.0–10	22	40.0	6.6 ± 0.6	
	11.0–15	11	20.0	8.5 ± 0.3	
	> 15	12	21.8	10.6 ± 0.8	

**Table 3:** Medicinal plants used for respiratory tract disorders in Urambo District.

**Scientific name**	**Local name**	**OG**	**LF**	**PU**	**So**	**Ailment cured**	**Preparation and administration**	**RFC**	**UV**	**FIV**	**CS**
Amaranthaceae										20	
*Chenopodium opulifolium* Schrad. ex W.D.J.Koch & Ziz (UR39)	Mwoshafedha	N	H	L	W	Influenza, cold	Decoction drunk, steam inhalation	0.200	0.182		—
Anacardiaceae										15	
*Mangifera indica* L. (UR26)	Mwembe	I	T	Se, L	C	Coughs, colds, asthma	Decoction drunk	0.145	0.291		DD
Annonaceae										24	
*Hexalobus monopetalus* (A.Rich.) Engl. & Diels (UR19)	Mkuwa	N	T	L	W	Colds, coughs, bronchitis	Decoction drunk	0.236	0.382		LC
Apocynaceae										33	
*Diplorhynchus condylocarpon* (Müll.Arg.) Pichon (UR32)	Msongati	N	S	R	W	Chronic coughs, pneumonia, tuberculosis	Concoction drunk	0.109	0.164		LC
*Strophanthus eminii* Asch. ex Pax. (UR27)	Mvelevele	N	T	R	W	Influenza, coughs	Decoction drunk	0.218	0.418		—
Asphodelaceae										38	
*Aloe vera* (L.) Burm.f. (UR29)	Mlovera	I	H	Wh	W	COVID-19, sore throat, coughs, asthma	Peeled leaves are eaten to relieve sore throat and cough. Infusion is drunk for asthma	0.382	0.255		—
Asteraceae										13	
*Ageratum conyzoides* L. (UR07)	Msendawazi	I	H	R, F	W	Asthma, coughs, colds	Infusion of powdered roots is drunk for asthma. Flowerheads mixed with *Ocimum tenuiflorum* are used for coughs and cold	0.127	0.182		LC
Caricaceae										11	
*Carica papaya* L. (UR37)	Mpapai	I	T	L, F	C	COVID-19, bronchitis	Decoction of young leaves drunk for treating COVID-19. Infusion of flowers is drunk for bronchitis	0.109	0.145		DD
Combretaceae										9	
*Combretum pisoniiflorum* (Klotzsch) Engl. (UR31)	Mlama	N	T	B	W	Asthma	Crush when mixed with salt	0.091	0.091		—
Cucurbitaceae										26	
*Momordica charantia* L. (UR25)	Umotomoto	N	H	L	W, C	Asthma, tuberculosis	Decoction drunk	0.255	0.382		—
Euphorbiaceae										40	
*Ricinus communis* L. (UR34)	Mnyonyo	I	S	L, R	W	Coughs	Decoction drunk	0.255	0.236		—
*Spirostachys africana* Sond. (UR04)	Mtomboti	N	T	B	W	Influenza	Chew and swallow the juice	0.145	0.145		LC
Fabaceae										**84**	
*Cassia abbreviata* Oliv. (UR35)	Muzoka	N	T	B	W	Pneumonia	Decoction drunk	**0.527**	0.309		LC
*Delonix elata* (L.) Gamble (UR17)	Mutangala	N	S	R	W, C	Coughs, asthma	Decoction drunk	0.200	0.327		LC
*Dichrostachys cinerea* (L.) Wight & Arn. (UR38)	Mkulagembe	N	S	L	W, C	Coughs	Infusion drunk	0.073	0.073		LC
*Entada abyssinica* Steud. ex A.Rich. (UR33)	Mfutwamila	N	S	B	W, C	Colds, coughs	Decoction drunk	**0.527**	**0.673**		LC
*Erythrina abyssinica* Lam. (UR24)	Mlinzi	N	T	B, R	W, C	Asthma	Decoction of pounded parts drunk	0.091	0.091		LC
*Piliostigma thonningii* (Schumach.) Milne-Redh. (UR18)	Mkindwambogo	I	T	Fr	W	Coughs	Chew and swallow the juice	0.091	0.109		—
*Vachellia nilotica* (L.) P.J.H.Hurter & Mabb. (UR30)	Mdubilo	N	T	B	W	Asthma, coughs	Decoction drunk	0.182	0.309		LC
Lamiaceae										18	
*Clerodendrum myricoides* (Hochst.) R.Br. ex Vatke. (UR36)	Mpugambu	N	T	R	W	Asthma, coughs	Powdering of roots	0.182	0.200		EN
Loganiaceae										46	
*Strychnos mitis* S.Moore (UR15)	Mwangajini	N	T	L	W	Whooping coughs	Decoction drunk	0.291	0.291		—
*Strychnos potatorum* L.f. (UR13)	Mgwegwe	N	T	R, L, Se	W	Coughs, bronchitis,	Decoction of roots and leaves treated coughs, while that of seed is drunk for bronchitis	0.200	0.364		—
Meliaceae										24	
*Trichilia emetica* Vahl (UR05)	Mtimaji	N	T	R, B	W, C	Pneumonia, colds	Decoction drunk	0.236	0.236		LC
Menispermaceae										36	
*Cissampelos pareira* L. (UR11)	Mkulawanti	N	H	Rh	W	Coughs	Decoction drunk	0.364	0.273		—
Moraceae										16	
*Ficus thonningii* Blume (UR03)	Mlundalunda	N	T	L	W, C	Colds, sore throat, tuberculosis	Decoction drunk	0.164	0.455		LC
Musaceae										6	
*Musa paradisiaca* L. (UR09)	Mgomba	I	H	L	C	Colds, bronchitis	Dried leaves made into syrup and drunk	0.055	0.109		—
Myrtaceae										**62**	
*Eucalyptus globulus* Labill. (UR20)	Mkalatusi	I	T	L	W, C	COVID-19, bronchial complaints	Steam inhalation	0.309	0.509		LC
*Psidium guajava* L. (UR28)	Mpera	I	T	L	C	Coughs, influenza	Infusion drunk	0.127	0.255		LC
*Syzygium aromaticum* (L.) Merr. & L.M.Perry (UR21)	Mkarafuu	I	T	S	C	Coughs, influenza	Powdering then mix with tea	0.327	**0.655**		**—**
Olacaceae										33	
*Ximenia caffra* Sond. (UR01)	Mtundadamu	N	T	L, R	W, C	Coughs, tonsillitis	Extracts of leaves are gargled for soothing tonsillitis. Infusion of roots combined with leaves is drunk for coughs	0.327	0.564		LC
Oleaceae										13	
*Schrebera trichoclada* Welw. (UR41)	Mputika	N	T	L	W	Coughs, influenza	Chew and swallow the juice	0.127	0.218		LC
Pedaliaceae										15	
*Sesamum angolense* Welw. (UR23)	Mulendagwawima	N	H	R	W, C	Coughs, colds	Infusion drunk	0.145	0.273		—
Phyllanthaceae										44	
*Bridelia micrantha* (Hochst.) Baill. (UR40)	Mtemela	N	T	R	W	Coughs	Infusion drunk	0.291	0.218		LC
*Phyllanthus amarus* Schumach. & Thonn. (UR16)	Mbondo	I	H	Wh	W, C	Coughs	Decoction combined with honey is drunk	0.164	0.164		—
Rhamnaceae										15	
*Ziziphus mucronata* Willd. (UR14)	Kagowole	N	T	B	W, C	Coughs	Infusion drunk	0.145	0.091		LC
Rubiaceae										35	
*Crossopteryx febrifuga* (Afzel. ex G.Don) Benth. (UR22)	Msasambeke	N	T	R	W, C	Coughs, influenza	Concoction drunk	0.109	0.182		LC
*Vangueria infausta* Burch. (UR12)	Mfulara	N	S	R	W, C	Pneumonia, coughs	Warm decoction drunk	0.273	0.545		LC
Rutaceae										47	
*Citrus aurantifolia* (Christm.) Swingle (UR08)	Mndimu	I	T	L	C	Asthma	Decoction mixed with honey and egg, then drunk	0.345	0.218		LC
*Citrus limon* (L.) Burm.f (UR10)	Mlimao	I	T	Fr	C	Coughs, cold	Decoct, then mix with *Allium sativum*	0.473	0.309		LC
Sapindaceae										22	
*Zanha africana* (Radlk.) Exell. (UR02)	Mkalya	N	T	R, B	W	Colds, tonsillitis	Decoction of roots drunk while the powder of bark is used as snuff	0.218	0.491		—
Solanaceae										9	
*Solanum incanum* L. (UR42)	Ndulele	N	H	R	W, C	Coughs, sore throat	Chew or swallow the sap	0.091	0.127		LC
Zingiberaceae										**66**	
*Zingiber officinale* Roscoe (UR06)	Tangawizi	I	H	Wh	C	Coughs, influenza, cold, throat infection	Decoction drunk	**0.655**	**0.618**		DD

*Note:* Bold numbers represent dominance in terms of FIV, RFC, and UV.

Abbreviations: B, bark; C, cultivated; CS, conservation status; DD, data deficient; EN, endangered; F, flower; FIV, family importance value; Fr, fruit; H, herb; I, introduced; L, leaf; LC, least concern; LF, life form; N, native; OG, origin; PU, parts used; R, root; RFC, relative frequency of citation; Rh, rhizome; S, shrub; Se, seed; So, source; T, tree; UV, use value; W, wild; Wh, whole plant.

**Table 4:** Informant consensus factor for each ailment type.

**Ailment type**	**Number of use reports (Nur)**	**Number of species (Nt)**	**Informant consensus factor (ICF)**
Coughs	361	29	**0.922**
Flu	87	8	**0.919**
Asthma	101	10	**0.910**
Pneumonia	28	4	0.889
Colds	71	9	0.886
Sore throat	18	3	0.882
Tonsillitis	9	2	0.875
COVID-19	17	3	0.875
Bronchitis	33	5	0.875
Tuberculosis	8	3	0.714

*Note:* Bold numbers represent RTDs with highest ICF.

**Table 5:** Comparison between the present study and other ethnomedicinal studies on RTDs in other African countries.

**Study area**	**Country**	**Total recorded MPs**	**Total MPs in the present study**	**Number of common MPs**	**JI**	**Source**
Maritime	Togo	98	42	12	7.9	[64]
Osum State	Nigeria	87	42	8	5.8	[65]
Maputaland	South Africa	30	42	4	5.3	[66]
Kamuli, Kisoro, and Nakapiripirit	Uganda	88	42	7	5.1	[16]
Kisumu	Kenya	44	42	4	4.4	[40]
Oyo State	Nigeria	26	42	3	4.2	[61]
Oran	Algeria	65	42	4	3.6	[67]
Oum Rbai	Morocco	66	42	3	2.7	[68]

## Data Availability

The arithmetical information used to support the findings of this study is available from the corresponding author upon request.
